# Silicone Rubber Catheters Modified by Poly(N-vinylpyrrolidone) Graft Promoted by Gamma Rays

**DOI:** 10.3390/polym17050600

**Published:** 2025-02-24

**Authors:** Jesús Enrique López-Meza, Víctor Hugo Pino-Ramos, Guadalupe Gabriel Flores-Rojas, Eduardo Mendizabal, Emilio Bucio

**Affiliations:** 1 Departamento de Química de Radiaciones y Radioquímica, Instituto de Ciencias Nucleares, Universidad Nacional Autónoma de México, Circuito Exterior, Ciudad Universitaria, Mexico City 04510, Mexico; jesus.lopezma@udlap.mx (J.E.L.-M.); vickor_ari@hotmail.es (V.H.P.-R.); 2 Departamento de Química, Centro Universitario de Ciencias Exactas e Ingenierías, Universidad de Guadalajara, Blvd. M. García Barragán #1451, Jalisco 44430, Mexico; lalomendizabal@hotmail.com

**Keywords:** N-vinylpyrrolidone, graft polymerization, gamma rays, silicone rubber, catheters

## Abstract

The functionalization of polymeric matrices through graft polymerization offers multiple advantages by introducing new properties through the incorporation of functional chemical groups on the surface. These modifications can change the chemical properties of the matrix, such as hydrophilicity and chemical reactivity, and enable new chemical interactions with other molecules that the matrix alone could not achieve. This expands the applicability of the material in various fields, particularly in medicine, where these functionalized matrices can be employed as drug delivery systems. In this work, poly(N-vinylpyrrolidone) was incorporated through a graft polymerization of N-vinylpyrrolidone on silicone rubber catheters using gamma radiation to promote the polymerization reaction on the matrix. The graft degree of poly(N-vinylpyrrolidone) on the SR matrix was studied based on the absorbed dose and monomer concentration. Additionally, the new materials were characterized using TGA, wettability kinetics, ATR-FTIR, and Raman spectroscopy, as well as a drug loading evaluation.

## 1. Introduction

The growing need to develop materials with specific properties, such as the controlled release of therapeutic agents and the inhibition of the proliferation of microorganisms on their surfaces, has become highly relevant today, especially in materials used in the medical field, where they are used as part of treatments for wound healing and systems responsible for protecting against bacterial infections. These considerations have significantly impacted the medical sector, driving the development of new polymeric materials that provide innovative solutions through the loading and release of therapeutic and antimicrobial agents.

In this sense, silicone rubber is a polymeric material widely used in the medical area due to its excellent biocompatibility, thermal resistance, chemical stability, and flexibility [1,2,3]. These characteristics make it suitable for medical applications that require interactions with biological tissues, such as prosthetics, catheters, implants, and medical seals [2,4,5,6]. However, for certain specialized applications, it is necessary to enhance the properties of silicone rubber, such as its hydrophilicity, moisture retention capacity, or resistance to bacterial growth. This has driven the development of modifications to its matrix using functional monomers [7].

One of the most promising monomers in the field of medicine is N-vinylpyrrolidone (NVP) due to the unique properties of its resulting polymer, poly(N-vinylpyrrolidone) (PNVP). This polymer is widely known for being biocompatible [8], hydrophilic [8], and nontoxic [9], making it an ideal component in medical applications such as coatings for medical devices, drug delivery systems, membranes, and contact lenses [10,11,12]. The modification of silicone rubber matrices with PNVP allows for additional properties such as increased hydrophilicity, moisture retention capacity, and load of potential antimicrobial agents, significantly enhancing their performance in medical environments [13].

Gamma radiation-induced graft polymerization has emerged as an efficient and controlled technique for modifying polymers without the need for chemical initiators. This process involves irradiating the base polymeric material (polymeric matrix) with gamma radiation (pre-irradiation method) or both the monomer and polymeric matrix (direct method), generating free radicals. These radicals subsequently react, promoting a graft polymerization onto the matrix. Unlike chemically induced grafting methods, gamma radiation offers significant advantages, such as minimizing toxic residues, precise process control, and the ability to carry out the reaction at room temperature using environmentally friendly solvents like water [14].

Several factors affect gamma radiation-induced graft polymerization, where the concentration of the monomer, degree of swelling or wettability of the matrix, and radiation dose are key factors in determining the grafting degree [14]. Some of the monomers that have been successfully grafted onto silicone rubber and other polymeric matrices using gamma radiation include acrylic acid (AAc), ethylene glycol dimethacrylate (EDGMA), glycidyl methacrylate (GMA), N-vinyl caprolactam (NVCL), and methyl methacrylate (MMA), among others [14,15,16]. The grafting of AAc onto polymeric matrices has increased hydrophilicity and imparted pH-responsive properties to the matrix [17], while the grafting of NVCL provides thermo-responsive properties [13]. These modifications expand the use of these advanced polymeric materials in applications such as catheters, implants, and medical membranes, where additional chemical resistance properties and biocompatibility are required.

Therefore, the modification of silicone rubber with PNVP using gamma radiation-induced graft polymerization offers an effective strategy to enhance the functional properties of this material in medical applications. The ability to control the concentration of the monomer, the radiation dose, and the degree of wettability of the material allows for the customization of the final product’s characteristics, expanding its potential use in innovative and advanced medical devices.

## 2. Materials and Methods

Silicone rubber (SR) catheters with a thickness of 1 mm, an inner diameter of 2.0 mm, and an outer diameter of 5.0 mm were sourced from Goodfellow. These catheters were cut into pieces measuring 5 cm, and then washed with methanol for 24 h and dried under vacuum until a constant weight was achieved. N-vinylpyrrolidone (NVP), obtained from Sigma Aldrich, was purified via vacuum distillation. Diclofenac sodium was obtained from Vorquímica, S.L. (Vigo, Spain). All solvents, including methanol and ethanol, were procured from J.T. Baker and used as received.

### 2.1. Modification of Silicone Catheters by Poly(N-vinylpyrrolidone) (SR-g-NVP)

SR catheters measuring 5 cm were placed inside an ampoule containing 7 mL of NVP with molar concentrations of 1 M, 2 M, and 2.5 M, and ethanol was used as the solvent. Subsequently, the oxygen from the air was removed from the samples using two different methods: the first involved degassing with argon for 10 min, and the second consisted of repeated freeze–thaw cycles using liquid nitrogen. The samples were then exposed to gamma rays from a ^60^Co source at various radiation doses at room temperature. Following this, the samples were thoroughly washed with ethanol to eliminate any homopolymer formed during the graft polymerization reaction. Finally, they were dried in a vacuum oven at 40 °C until a constant weight was achieved.

The graft percentage was calculated using the following equation (Equation (1)):

Graft (%) = 100((Wg − Wo)/Wo)(1)

where Wo and Wg correspond to the initial and final weights of the samples.

### 2.2. Characterization

#### 2.2.1. Structural Characterization Using ATR-IR and Raman Spectroscopy

Dry samples of both SR and SR-g-NVP catheters were examined using an ATR-FTIR Perkin-Elmer Spectrum 100 spectrometer (Norwalk, Connecticut, CT, USA) with 16 scans. The spectra were recorded over a range from 4000 to 650 cm^−1^. Raman spectra were recorded using a Renishaw inVia confocal Raman microscope (Renishaw Ibérica S.A.U., Barcelona, Spain). Data processing was carried out using the native software.

#### 2.2.2. Thermal Characterization

Samples of both SR and SR-g-NVP catheters were analyzed using TGA Q50 2010 TA instruments from New Castle, DE, USA. Thermal properties were recorded within a 25 to 800 °C temperature range using a heating rate of 10 °C min^−1^ under a nitrogen flow of 100 cm^3^ min^−1^.

#### 2.2.3. Wettability Capacity

The wettability properties of the catheters were evaluated in distilled water at 25 °C. The samples were initially dried until a constant weight was achieved and then immersed in water, and their weights were recorded at established time intervals. Excess water was gently removed using filter paper before weighing the samples. The wettability percentage was determined using the following equation (Equation (2)):

Wettability (%) = 100 ((Ws − Wd)/Wd)(2)

where Ws and Wd are the weights of the wet and dried catheters, respectively.

#### 2.2.4. Evaluation of Drug Loading

The loading of diclofenac was evaluated using SR-g-NVP 10% catheters with a size of 5 cm that had been previously washed and dried to a constant weight under vacuum conditions. The samples were immersed in vials containing 10 mL of an aqueous solution of diclofenac (36 μg/mL) under mechanical stirring. The loading process was monitored using UV spectrophotometry at room temperature, aliquots were taken from the loading medium at fixed time intervals to measure the absorbance at 276 nm, and a calibration curve was employed to determine the aliquots’ diclofenac concentration. After each measurement, the aliquots were returned to the loading medium. This experiment was conducted in triplicate to ensure the reproducibility of the results

The concentration of the drug loaded in the SR-g-NVP catheter was calculated using the following equation (Equation (3)):

Loaded drug (%) = (Df − Di)V/A(3)

where Di and Df are the initial and final concentrations of the loading medium in μg/mL at a specified time, V is the volume of the medium in ml, and A is the area of the sample in cm^2^.

## 3. Results and Discussion

### 3.1. Synthesis of SR-g-NVP

**Figure 1:**
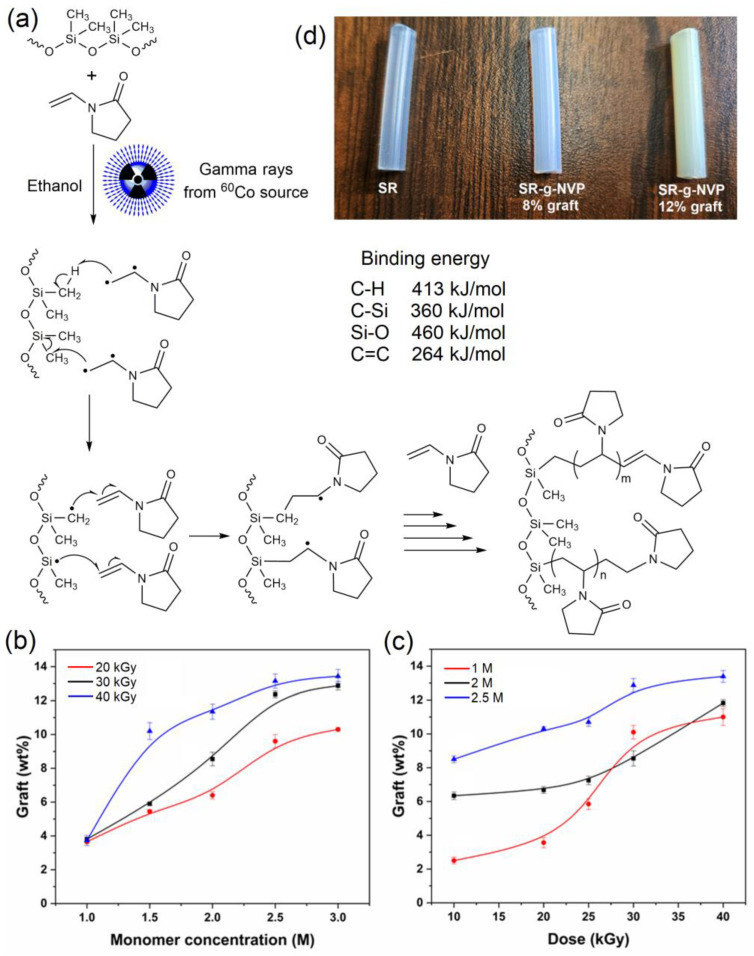
(**a**) Scheme of graft polymerization of PVP on SR catheters promoted by gamma rays. (**b**) Grafting degree as a function of monomer concentration at different doses (20 kGy, 30 kGy, and 40 kGy). (**c**) Grafting degree as a function of absorbed dose at different monomer concentrations (1 M, 2 M, and 2.5 M). (**d**) Photographs of the SR catheters with different graft percentages.

The SR catheters were successfully grafted with poly(N-vinylpyrrolidone) (PNVP) using gamma radiation to promote a radical polymerization that resulted in the grafting of PNVP polymer chains onto the film surface. The possible polymerization mechanism is shown in Figure 1a, without taking into consideration the effect of the ethanol used as a solvent. According to the bond energies, the probability of free radical formation is highest in N-vinylpyrrolidone (C=C, 264 kJ/mol). These free radicals can react with the silicone rubber matrix, generating new free radicals inside it. The formation of free radicals in the matrix originates from the C-H (413 kJ/mol) and Si-C (360 kJ/mol) bonds, which subsequently react with the monomer, initiating graft polymerization. The graft percentage in the catheters was studied as a function of monomer concentration and absorbed dose, which are critical factors in this process (Figure 1b,c). It was observed that an increase in the concentration of NVP and the absorbed dose resulted in an increase in the graft percentage; they reached a maximum of 12.8 ± 0.8% at a concentration of 2.5 M and an absorbed dose of 30 kGy and also caused a color change of the new material to brown (Figure 1d). However, at higher concentrations and doses, the increase was minimal, suggesting that elevated levels may induce undesirable reactions, such as homopolymerization of the monomer, degradation of the matrix, or polymer crosslinking, which could lead to a non-recovery of the modified catheter or a decrease in the mechanical properties of the final material.

On the other hand, an appropriate dose of gamma radiation is essential to optimize graft polymerization and avoid the degradation of the matrix polymer. These modifications can result in a significant increase in the degree of wettability of the matrix, which is beneficial for applications requiring high fluid retention. Additionally, the effect on graft percentage was evaluated using two methods of oxygen removal: argon bubbling and freeze–thaw cycles. The results demonstrated that degassing through argon bubbling allows for better control over the graft percentage.

### 3.2. Characterization

#### 3.2.1. Infrared Spectroscopy (ATR-FTIR) and Raman Spectroscopy

**Figure 2:**
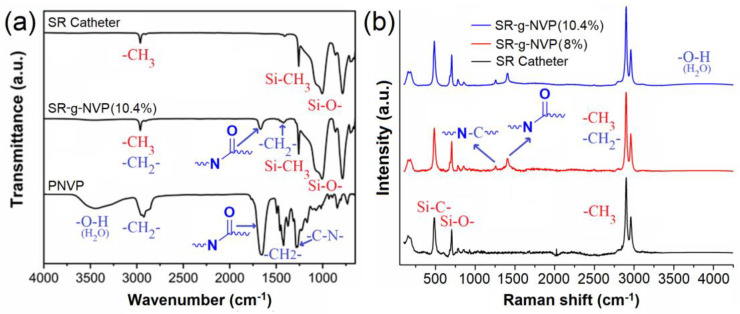
(**a**) Infrared spectra of SR catheter, SR–g–NVP 10.4% graft, and PNVP. (**b**) Raman spectra of SR catheter, SR–g–NVP 10.4% graft, and SR–g–NVP 8% graft.

The FTIR-ATR spectra of the pristine and modified catheters (SR and SR-g-NVP) are presented in Figure 2. The spectrum of the SR catheter showed bands corresponding to (ν) Si–O–Si bonds in the 1006–1073 cm^−1^ range, as well as a band at 1258 cm^−1^ assigned to (ν) Si–CH_3_ and another at 2963 cm^−1^ corresponding to (ν) C–H bonds from the polydimethylsiloxane matrix [18]. In the case of the SR-g-NVP catheters, the spectra revealed additional bands indicating the incorporation of the PNVP graft on the SR matrix. These bands include one assigned to the (ν, C=O) carbonyl group of the amide, corresponding to the pyrrolidone units, at 1672 cm^−1^, as well as bands associated with (ν) -CH_2_- bonds at 2975 and (δ) -CH_2_- bonds at 1420 cm^−1^ from the aliphatic chains generated during the graft polymerization of NVP. These bands were also observed in the PNVP spectrum, where an additional band corresponding to the (ν) C-N bonds at 1263 cm^−1^ from the pyrrolidone units and another assigned to the (ν) O-H bonds at 3412 cm^−1^, attributed to moisture in the PNVP, were identified. These findings are consistent with previous reports on PNVP [19]. Furthermore, it was observed that the intensity of the bands associated with the grafted polymer increased with the degree of PNVP grafting on the SR matrix. A more detailed analysis was carried out by means of Raman spectroscopy of the catheters (Figure 2b), where the spectrum of copolymers exhibited a band at 2818–2956 cm^−1^ that corresponded to the symmetrical and asymmetrical stretching (ν) of C-H bonds from the methyl and aliphatic units from the silicone rubber and grafted PNVP chains, at 1410 cm^−1^ due to the band of (ν) C=O bonds from the pyrrolidone units of the grafted PNVP, at 1260 cm^−1^ owing to (ν) C-N bonds, and at 706 cm^−1^ and at 494 cm^−1^ due to (ν) Si-C bonds and (ν) Si-O bonds.

#### 3.2.2. Thermal Characterization

**Figure 3:**
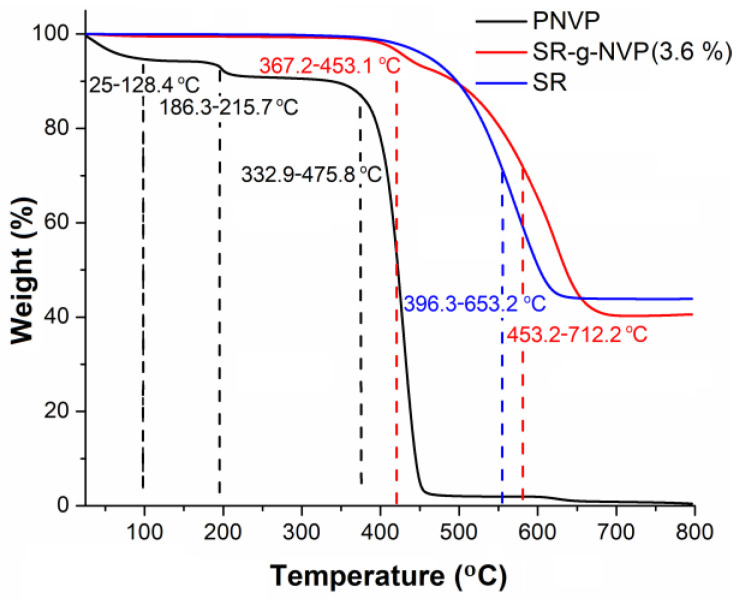
TGA analysis of the SR catheters and catheter modified by PNVP grafting (SR–g–NVP) with a graft degree of 3.6%. The analysis was conducted under nitrogen flow and 10 °C/min.

Thermogravimetric (TGA) studies revealed a weight loss of 10% for the homopolymer at 337.5 °C, which was in contrast to a 10% weight loss at 494.2 °C for the SR–g–NVP; this thermal stability was provided by the SR, which was responsible for this weight loss at 496.9 °C. The homopolymer exhibited an initial weight loss in the 25–128.4 °C range, which was attributed to moisture (5.7%) present in the PNVP chains. This was followed by a second weight loss step from 186.3 to 215.7 °C, with a mass loss of approximately 3.6%, corresponding to volatile compounds remaining in the homopolymer, and finally, the degradation of the PNVP homopolymer, which began with the formation of one of the primary degradation compounds, pyrrolidone, as well as the formation of a polymeric chain or, alternatively, aliphatic and aromatic hydrocarbons of low molecular weight. This process occurred within a temperature range from 332.9 to 475.8 °C, resulting in a total weight loss of 88.8% [20] (Figure 3).

For the SR-g-NVP catheters, the decomposition of the PNVP graft occurred in a range from 367.2 to 453.1 °C, indicating a higher thermal stability compared to the homopolymer obtained using this methodology (332.9–475.8 °C), which presented a decomposition temperature similar to commercial PNVP [21]. This behavior is attributed to the formation of copolymers with the SR matrix, which enhances thermal stability and reduces the moisture absorption capacity of the PNVP graft polymer from the environment. This reduction in moisture absorption is primarily due to the hydrophobic properties of the SR matrix [22]. The TGA study supports this observation, showing no significant weight loss at temperatures up to 128.4 °C. Finally, the SR-g-NVP exhibited a final decomposition temperature in a range from 453.2 to 712.2 °C, which corresponds to the SR matrix, and a higher thermal stability compared to the pristine SR matrix that decomposed in a range from 396.3 to 653.2 °C. This decomposition temperature is comparable to previously reported values for silicone rubber films [14]. In addition, a residue of 38.3% was observed at 800 °C, primarily corresponding to SiO and SiO_2_, as well as ashes from the SR-g-NVP catheter [23,24].

#### 3.2.3. Wettability Degree

The wettability tests allowed for a detailed examination of the affinity of the new materials for water, a crucial factor in the biocompatibility of materials intended for medical purposes [25]. The SR-g-NVP catheters were immersed in distilled water at room temperature, showing an increase in their degree of wettability until reaching a maximum value, at which point their water absorption capacity was exhausted.

**Figure 4:**
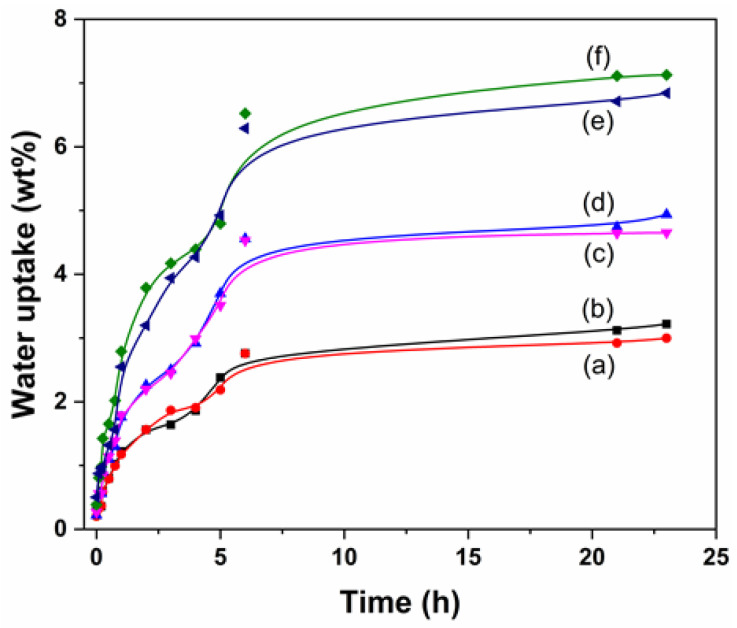
Wettability samples in distilled water at 25 °C. The samples were obtained under conditions with different concentrations and gamma ray doses. (a) SR-g-NVP 3.6% graft, 1M, 40 kGy; (b) SR-g-NVP 3.8% graft, 1M, 30 kGy; (c) SR-g-NVP 6.6% graft, 2M, 10 kGy; (d) SR-g-NVP 6.5% graft, 2M, 20 kGy; (e) SR-g-NVP 10% graft, 2M, 40 kGy; and (f) SR-g-NVP 10% graft, 2M, 20 kGy.

Figure 4 illustrates the wettability kinetics of the catheters, highlighting an increase in the degree of wettability with the increase in PNVP grafting. This is because the SR matrix is hydrophobic, and its modification reduces repulsion towards water. The SR-g-NVP catheter with a graft percentage of 3.6% showed a low wettability percentage of 2.9%, while the catheters with graft percentages of 10% exhibited a more notable change, absorbing most of the water during the first 5 h of immersion and reaching equilibrium within 20 h, with a maximum wettability of 6.9%. This behavior is similar to that observed in the previous report of catheters grafted with 10% N-vinylimidazole (NVI), suggesting that increasing the degree of grafting can enhance the wettability percentage [24].

Although, in general, the graft percentage increased the wettability percentage, it could also be affected by the absorbed dose, as indicated in samples e and f in Figure 4. The sample with an absorbed dose of 40 kGy (Figure 4e) exhibited a lower wettability percentage compared to the sample obtained with a dose of 20 kGy (Figure 4f). This behavior can be attributed to crosslinking promoted by the excess energy provided after the polymerization reaction consumed the monomer.

### 3.3. Drug Loading Test

Once the catheters (SR-g-NVP 10%) were obtained and characterized, tests were conducted to evaluate their capacity to load diclofenac as a therapeutic agent. The results demonstrated that the SR-g-NVP catheters have the ability to incorporate this drug, resulting in a maximum loading of 11.01 µg/cm^2^ over a period of 6 h. These findings indicate that the grafting of PNVP significantly improved the physicochemical properties of the SR-based catheter by providing a surface with hydrophilic character and functional groups that facilitate chemical interactions with the molecules of interest. This enhancement was also observed in the previous report of catheters that were grafted with NVI and loaded with ampicillin, a broad-spectrum antibiotic, resulting in a maximum loading of 654 µg/cm^2^ with a grafting degree of 39% and a release sustained for several days [24]. These findings indicate that it is possible to increase the drug loading in relation to the grafting percentage, and, unequivocally, the grafting polymerization can confer favorable physicochemical properties to the silicone catheters, facilitating the loading and release of therapeutic agents, which could enhance their applications as medical devices.

## 4. Conclusions

The graft copolymerization of PNVP on SR catheters was successfully carried out using the direct irradiation method. Precise control of the graft percentage was achieved by manipulating the absorbed dose and the concentration of the monomer, resulting in a graft percentage ranging from 2.6% to 12.8%. These factors not only regulate the graft percentage but also allow for control over the formation of homopolymer and the degradation of the matrix.

The modification of SR catheters with PNVP (SR-g-NVP) caused significant changes in the wettability properties of the matrix, transforming it from hydrophobic to hydrophilic. This transformation was evidenced by a maximum wettability of 6.9%. In conclusion, these results pave the way for new materials with enhanced hydrophilic properties and functional groups that enable the loading of therapeutic agents such a diclofenac with a load of 11.01 µg/cm^2^, which may expand the potential applications of the SR-g-NVP catheter as a medical device.

The use of gamma radiation eliminates the need for toxic chemical agents and allows for work under controlled environmental conditions, making this method a more sustainable and less polluting option compared to other polymer functionalization processes.

The improvements achieved in this study position SR-g-PNVP catheters as promising candidates for advanced medical applications, such as antibacterial catheters, drug delivery devices, or implants that interact more effectively with biological tissues. This study lays a solid foundation for the development of innovative medical devices with customizable properties that could significantly impact the fields of regenerative medicine and therapy administration.

## Data Availability

The original contributions presented in the study are included in the article, further inquiries can be directed to the corresponding author.
